# An emergency department medical record review for adolescent intentional self-harm injuries

**DOI:** 10.1186/s40621-020-00293-8

**Published:** 2021-01-08

**Authors:** Anna Hansen, Dessi Slavova, Gena Cooper, Jaryd Zummer, Julia Costich

**Affiliations:** 1 Kentucky Injury Prevention and Research Center, University of Kentucky, Lexington, KY USA; 2 Department of Sociology, University of Kentucky, Lexington, USA; 3 College of Medicine, University of Kentucky, Lexington, KY USA; 4 Department of Emergency Medicine, College of Medicine, University of Kentucky, Lexington, KY USA; 5 Department of Health Management & Policy, College of Public Health, University of Kentucky, 111 Washington Ave, Lexington, KY 40536-0003 USA

**Keywords:** Intentional self-harm, Adolescence, Suicide, Population surveillance, ICD-10-CM

## Abstract

**Background:**

Non-suicidal self-injury and suicide attempts are increasing problems among American adolescents. This study developed a definition for identifying intentional self-harm (ISH) injuries in emergency department (ED) records coded with International Classification of Diseases, 10th Revision, Clinical Modification (ICD-10-CM) codes. The definition is based on the injury-reporting framework proposed by the Centers for Disease Control and Prevention. The study sought to estimate the definition’s positive predictive value (PPV), and the proportion of ISH injuries with intent to die (i.e., suicide attempt).

**Methods:**

The study definition, based on first-valid external cause-of-injury ICD-10-CM codes X71-X83, T14.91, T36-T65, or T71, captured 207 discharge records for initial encounters for ISH in one Kentucky ED. Medical records were reviewed to confirm provider-documented diagnosis for ISH, and identify intent to die or suicide ideation. The PPV of the study definition for capturing provider-documented ISH injuries was reported with its 95% confidence interval (95% CI).

**Results:**

The estimated PPV for the study definition to capture ISH injuries was 88.9%, 95% CI (83.8%, 92.8%). The estimated percentage of ISH with intent to die was 45.9, 95% CI (47.1, 61.0%). The ICD-10-CM code “suicide attempt” (T14.91) captured only 7 cases, but coding guidelines restrict assignment of this code to cases in which the mechanism of the suicide attempt is unknown.

**Conclusions:**

The proposed case definition supported a robust PPV for ISH injuries. Our findings add to the evidence that the current ICD-10-CM coding system and coding guidelines do not allow identification of ISH with intent to die; modifications are needed to address this issue.

## Background

Intentional self-harm (ISH) and attempted suicide are increasingly prevalent among adolescents in the United States.(Miron et al. 2019) ISH is purposeful harm towards one’s own body, and includes injuries such as poisoning, cutting, burning, and scratching. ISH is associated with psychiatric distress and risk of suicide.(Wilkinson 2011) ISH is common among youth, with onset between ages 12–14. (Cipriano et al. 2017) Reported rates of ISH among adolescents range widely from 7.5 to 46.5% in different samples, and appear to be increasing (Cipriano et al. 2017; Peterson et al. 2008). Suicide attempts (SAs) are non-fatal self-injurious behaviors with the intent to die.(Suicide 2020) Data concerning the prevalence of suicide attempts among adolescents remain limited. In 2019, the Centers for Disease Control and Prevention documented 2039 suicide deaths among adolescents 14–18 years of age, making it the second leading cause of death within this age group.(Ivey-Stephenson 2020) Suicide mortality mirrors trends in ISH and SA: from 2007 to 2017, the rate of suicide deaths among individuals aged 15–19 in the United States nearly doubled.(Miron et al. 2019)

Although non-suicidal ISH and SA are associated, each introduces distinct challenges concerning assessment, documentation and clinical management among adolescents. Clinical assessment of self-injuries may be complicated by patients’ impulsivity, non-disclosure, poor physician-patient rapport,(Cerel et al. 2006) and somnolence attributable to drug overdose. Assessment may be further impeded by comorbid diagnoses associated with self-harm such as attention deficit hyperactivity disorder (ADHD), autism, and intellectual or developmental disabilities. Documentation of the patient’s self-reported injury intent is essential for clinical assessment of ISH and SA. Accurate documentation of SAs is particularly crucial, as a history of SA is strongly associated with future suicide death.(Joiner and Rudd 2000) Although ISH is considered a significant risk factor for future suicide regardless of suicidal intent,(Hawton et al. 2003) a history of SA confers a higher risk for suicide than non-suicidal ISH.(Chan et al. 2016) In sum, accurate detection and documentation of patients’ suicidal intent is critical to understanding their risk of subsequent suicide. Patients’ needs for hospital admission, crisis management, and psychiatric care will differ based on whether they experienced non-suicidal ISH or SA.

The assessment of ISH and SA is further complicated by adolescents’ impulsivity. One study found nearly half (47.6%) of patients reported an interval of 10 min or less between the onset of suicidal thoughts and their SA.(Deisenhammer et al. 2009) The detection of suicidal ideation may be a means to identify SAs. However, if suicidal ideation is not identified because of a patient’s impulsivity (i.e. the patient denies ideation because they claim an injury was an act of impulse), challenges arise concerning the accurate assessment of the injury and surveillance of self-harm.

It is important to note differences between psychiatric and injury surveillance terminology for suicide and self-harm. Psychiatric terminology alludes to the intended outcome of the act (“non-suicidal” versus “suicide attempt”). Epidemiological injury surveillance terminology relies on coded discharge diagnoses in the administrative billing records. The current coding system, International Classification of Diseases, 10th Revision- Clinical Modification (ICD-10-CM),(National Center for Health Statistics 2016) does not distinguish between ISH with and without lethal intent. While there is a code labeled “suicide attempt” (T14.91), the ICD-10-CM coding guidelines specify that this code may only be assigned when the nature and body region of injury and the mechanism of the suicide attempt are not known.(Hedegaard et al. 2018) For some injury mechanisms (e.g., poisoning or suffocation), the intentionality of the injury is embedded in the diagnostic code (a new concept introduced in ICD-10-CM)(National Center for Health Statistics 2016). For example, the 6th character “2” of the code T42.4X2A indicates that the benzodiazepine poisoning was ISH. In other injury mechanisms, a separate external cause-of-injury (ECI) code is used to describe both the mechanism and intent (e.g., X78.1XX describes an ISH injury by knife that is included in the mechanism category of cut/pierce). The National Center for Health Statistics maintains the classification of ICD-10-CM codes by mechanism and intent (ICD–10–CM External Cause-of-Injury Matrix),(Hedegaard et al. 2019) and provides annual updates on appropriate code usage.(National Center for Health Statistics 2019) The code labeled “suicide attempt” (T14.91) appears in the unspecified self-harm category in the ICD–10–CM External Cause-of-Injury Matrix.

Hedegaard et al. provide insight into challenges of medical documentation and coding of ISH that should be considered when establishing a surveillance case definition. The authors also note that “in developing a surveillance case definition for hospitalizations and ED visits for suicide attempts and ISH, consideration should be given to testing the ability of the surveillance case definition to identify true cases.”(Hedegaard et al. 2018)

Our study proposed a definition for capturing ISH injuries in ICD-10-CM-coded ED discharge data that is aligned with both the methodology for identifying *injury* encounters in ED discharge data proposed by the Centers for Disease Control and Prevention (CDC)(Hedegaard et al. 2017) and the proposed ICD-10-CM External Cause Matrix grouping(Hedegaard et al. 2018; National Center for Health Statistics 2019; Hedegaard et al. 2017) of ICD-10-CM codes to identify specific ISH mechanism codes. We selected a representative sample of ED billing records from one Kentucky facility that were identified by the study definition as ED records for treatment of ISH injuries among pediatric patients. We reviewed the medical records for the identified cases to verify if the medical record for the treatment encounter included provider-documented information for an ISH injury. The first goal of the study was to estimate the positive predictive value (PPV) of the study surveillance definition for capturing provider-documented ISH injuries. The second goal was to estimate the proportion of the sampled ISH injury ED records with provider documentation for attempted suicide (i.e. ISH injury with intent to die). Knowing the limitations of the ICD-10-CM coding guidelines, we expected the number of ISH injury records with provider-documented suicide attempt to be much higher than those captured by T14.91.

## Methods

**Fig. 1:**
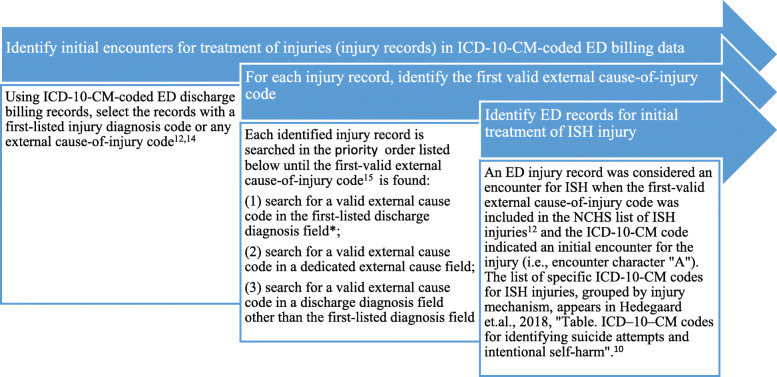
Proposed study definition for identifying emergency department discharge billing records for initial treatment of intentional self-harm (ISH) injuries in records coded with the International Classification of Diseases, 10th Revision, Clinical Modification (ICD-10-CM). *The term “first-listed discharge diagnosis field” in ED billing records is analogous to “principal diagnosis” for inpatient discharge records

The proposed study definition for capturing ISH injuries in ICD-10-CM-coded ED discharge billing records is described in Fig. 1. ED discharge administrative records for initial treatment of injury were identified based on a first-listed injury diagnosis code or any mention of an external cause-of-injury code, following the proposed CDC definition for identifying injury records in ICD-10-CM-coded ED discharge billing data.(National Center for Health Statistics 2019; Hedegaard et al. 2017) Then, for each injury ED record, a first-valid external cause-of-injury code(ISW9 2016) was identified by searching the record in a priority order for: (1) a valid external cause code in the first-listed discharge diagnosis field; (2) a valid external cause code in a dedicated external cause field; or (3) a valid external cause code in a discharge diagnosis field other than the first-listed diagnosis field. For example, if an ED record contained a first-listed diagnosis T43.632A (poisoning by methylphenidate, intentional self-harm, initial encounter) and a code X78.8XXA (intentional self-harm by other sharp object, initial encounter) in a dedicated external cause field, the code T43.632A would be selected as the first-valid external cause-of-injury code.

An ED record was considered an encounter for ISH injury when the first-valid external cause-of-injury code was included in the National Center for Health Statistics list of ISH injuries(National Center for Health Statistics 2019) and the ICD-10-CM code indicated an initial encounter for the injury (i.e., encounter character “A”). The list of specific ICD-10-CM codes for ISH injuries, grouped by injury mechanism, is described in Hedegaard et al. 2018.(Hedegaard et al. 2018) The study definition identified a sample of 207 records for ISH injuries for pediatric patients (age < 18 years) treated at an academic health center’s EDs, with discharge dates between January 1, 2016 and September 30, 2019. Patients admitted from an ED to the same acute care hospital appear in the administrative records as inpatient rather than ED discharges. The records included in this sample therefore included patients who were discharged home from the ED, left against medical advice, died in the ED, or were transferred to other facilities, including psychiatric hospitals, rehabilitation centers, and other acute care hospitals.

The medical records for the identified 207 billing records for initial ED treatment of ISH injuries were reviewed to confirm provider-documented diagnosis for ISH and to collect additional information on documented intent to die (i.e. suicide attempt), as well as documentation of suicidal ideation.

A data abstraction form was developed with input from two pediatric emergency medicine physicians, an adolescent medicine physician, and injury epidemiologists. The form was used to collect information on the documented presence of injury, the mechanism and intent of injury, and the presence of risk factors associated with self-harm behavior as documented in responses to the Patient Health Questionnaire-9.(Lowe et al. 2004) The abstract form included separate categories for documented ISH without intent to die (listed as “Intentional self-harm” for brevity), intentional self-harm with lethal intent (listed as “Suicide attempt”), and information on documented suicidal ideation. Two medical students reviewed the sampled records and abstracted data via Research Electronic Data Capture (REDCap), a secure, web-based application.(Harris et al. 2009)

The ICD-10-CM Official Guidelines for Coding and Reporting state that the “assignment of a diagnosis code is based on the provider’s diagnostic statement that the condition exists” and assignment “is not based on clinical criteria used by the provider to establish the diagnosis.”(Center for Medicare Services 2016) The coding guidelines further note that if medical record documentation is unclear or contradictory, the patient’s attending provider should be queried for clarification. The study team could not verify whether medical coders queried the providers for additional clarification when the attending provider’s notes were not complete or specific enough for assigning injury intent. For this reason, we estimated the PPV of the study definition for capturing provider-documented ISH in two ways:

1. based on the attending physician’s notes only (conservative PPV), and
2. based on the information in the entire medical record for the specific encounter of care.

The PPV of the study definition was first estimated as the proportion of cases confirmed by the study reviewers as provider-documented ISH injuries (with or without lethal intent) based on: (1) the attending physician’s notes only (study reviewers responded to the question “There is sufficient documentation by the ED attending to confirm the following” by selecting either “Intentional self-harm” or “Suicide attempt”) and (2) the information in the entire medical record associated with this encounter of care (the study reviewers responded to the question “There is sufficient documentation within the medical record for this encounter to confirm the following” by selecting either “Intentional self-harm” or “Suicide attempt”). A sub-analysis was completed to estimate the difference in prevalence of risk factors associated with self-harm behaviors between the group of ISH cases with documentation of suicidal intent in the provider notes (e.g. suicide attempts based on reviewer assessment), and the group of ISH cases without documentation of suicidal intent. A chi-square test was used to assess the statistical significance of differences in prevalence between the two groups.

Statistical analysis was performed using Stata v.15 (College Station, TX)(Stata Statistical Software [program] 2017) and SAS v.9.4 (Cary, NC) (SAS Institute Inc 2012). The estimated PPVs were reported with their exact 95% confidence intervals (95% CI). The Pearson chi-square test was used to compare the equality of proportions obtained from independent groups.

## Results

The study sample included 207 cases for ISH injuries among pediatric patients aged 4–17. Nearly half (*n* = 100) of the sampled cases were captured by codes for ISH with a drug poisoning mechanism (T36-T50) (Table 1). The second largest group (*n* = 82) was captured by codes for ISH injuries involving cutting or piercing. The 7 cases with unspecified mechanism were captured via the code T14.91 (suicide attempt).

**Table 1:** Frequency of the ICD-10-CM codes for identifying intentional self-harm injuries in the study sample

Code^a^	Mechanism	# of cases	% of total cases
T14.91	Unspecified	7	3.38
T36-T50	Drug poisoning	100	48.31
T54	Toxic effect of corrosive substances	< 5	–
T71	Asphyxiation due to hanging	< 5	–
X78	Cut/pierce	82	39.61
X79	Struck by/against	< 5	–
X80	Fall	< 5	–
X83	Other specified means	10	4.83
	Total	207	

^a^For additional information refer to Hedegaard H, Schoenbaum M, Claassen C, et al.(Deisenhammer et al. 2009)

The study reviewers found sufficient information in the attending physician’s notes to confirm 184 of the 207 sampled cases as ISH injuries (Table 2). Thus, the estimated PPV for the study definition’s ability to capture provider-documented ISH injuries was 88.9, 95% CI (83.8, 92.8%). There were 5 additional cases in which sufficient information for confirming an ISH was found outside the attending physician notes (e.g., psychiatric attending or behavioral health nurse), suggesting that the PPV could be as high as 91.3, 95% CI (86.6, 94.8%).

**Table 2:** Counts of confirmed provider-documented intentional self-harm, suicide attempt, and suicidal ideation in the study sample (*n* = 207) and estimated positive predictive value (PPV) and 95% confidence intervals for the study definitions

	Source: attending physician notes only			Source: all notes available in the medical record		
	Provider-documented cases (N)	Estimated PPV (%)	95% CI	Provider-documented cases (N)	Estimated PPV (%)	95% CI
Intentional self-harm	184	88.9^(a)^	(83.8, 92.8)	189	91.3^(b)^	(86.6, 94.8)
Intentional self-harm with intent to die (i.e. suicide attempt)	79	38.2	(31.5, 45.2)	95	45.9	(39.0, 52.9)
Suicidal ideation	106	51.2	(44.2, 58.2)	115	55.6	(48.5, 62.4)

(a) Estimated PPV for the study definition to identify provider-documented ISH injuries using attending physician notes only for the specific encounter of care

(b) Estimated PPV for the study definition to identify provider-documented ISH injuries using the documentation from attending physician and other providers/consults during the specific encounter of care

The attending physicians documented 79 (38.2%); 95% CI (31.5, 45.2%) cases as ISH injuries with intent to die (i.e., suicide attempt) (Table 2). An additional 16 cases were classified by the study reviewers as suicide attempts using information documented in notes from psychiatric consultations and behavioral health nurse assessments. Based on review of the entire encounter record, the estimated percentage of ISH ED discharge records with documented intent to die (i.e. suicide attempt) was 45.9, 95% CI (38.9, 52.0%).

More than half of the medical records documented suicidal ideation. Based on the review of the entire record for the sampled encounters of care, we estimated that 55.6% (95% CI (48.5, 62.4%)) of the ED discharge records for ISH documented patient expressions of suicidal ideation (Table 2). More than two-thirds of patients had histories of depressed mood disorder (*n* = 143, 69.1%) and mental health treatment (*n* = 144, 69.6%). The most commonly documented risk factors for ISH were relationship stressors and lack of social support (*n* = 138, 66.7%), school-related stressors (*n* = 108, 52.2%), bullying (*n* = 44, 21.3%), sexual abuse (*n* = 36, 17.4%), and physical abuse (*n* = 25, 12.1%) (Table 3).

**Table 3:** Estimated prevalence of risk factors associated with self-harm behaviors for the group of patients with (*n* = 95) and without (*n* = 112) provider-documented suicide attempt

Risk Factors	Cases with Provider Documentation for Suicide Attempt (***N*** = 95)		Cases Without Provider Documentation for Suicide Attempt (***N*** = 112)		***P***-value
	N	%	N	%	
Suicidal ideation	74	77.9%	41	36.61%	<.0001
History of depressed mood disorder	69	72.9%	74	66.07%	0.31
History of mental health treatment (medication/counseling)	69	72.9%	75	66.96%	0.38
History of substance abuse/dependence treatment	8	8.4%	11	9.82%	0.73
Crisis within last two weeks	11	11.6%	7	6.25%	0.18
Work-related stressors	1	1.1%	3	2.68%	0.40
School-related stressors	57	60.0%	51	45.54%	0.04
Relationship stressors/Lack of social support	75	78.9%	63	56.25%	0.001
Financial stressors	4	4.2%	4	3.57%	0.81
Patient is a victim of bullying	22	23.2%	22	19.64%	0.54
Patient is a victim of physical abuse	18	18.9%	7	6.25%	0.005
Patient is a victim of sexual abuse	20	21.1%	16	14.29%	0.20
Patient has a physical disability or health problem	3	3.2%	9	8.04%	0.13
Recent death of friend or family member (non-suicide)	14	14.7%	17	15.18%	0.93
Anniversary of a traumatic event	1	1.1%	1	0.89%	0.91
History of suicide attempts	33	34.7%	12	10.71%	<.0001
History of expressed suicidal thoughts	44	46.3%	32	28.57%	0.01
History of expressed suicidal plans	14	14.7%	9	8.04%	0.13
Legal/criminal problems	10	10.5%	11	9.82%	0.86
Patient is a current/recent prisoner	1	1.1%	0	0%	0.28
Housing instability/Homelessness	8	8.4%	11	9.82%	0.73
Family history of depression	40	42.1%	33	29.46%	0.06
Family history of suicide or suicide attempt	29	30.5%	12	10.71%	0.0004
Foster care	7	7.4%	10	8.93%	0.67
Recent move	6	6.3%	5	4.46%	0.55

The medical records indicated that 119 (57.5%) of the patients were currently in therapy, 132 (63.8%) were taking medications for mental/behavioral conditions, and 71 (34.3%) were on medications for other health conditions. Fifteen patients were maintained on 72-h hold; in another 106 cases a 72-h hold was ordered but then discontinued. In more than 90% of the cases there was documentation of a plan in place for treatment or follow-up service after discharge. Overall, 153 (73.9%) patients were discharged routinely to home/self-care but 23 of them were held initially for observation; the remaining 54 (26.1%) were discharged/transferred to psychiatric or other inpatient units.

Chi-square analyses demonstrated there were significant differences in the prevalence of risk factors associated with self-harm behaviors between those who had documented intention to die (i.e. attempted a suicide) and those who did not (Table 3). Adolescents who attempted suicide were more likely to report suicidal ideation (*p* < .0001), experience school-related stressor (*p* = 0.038), experience relationship stressors or lack of social support (*p* = 0.0006), be victims of physical abuse (*p* = 0.005), have a history of suicide attempts (p < .0001), have a history of expressed suicidal thoughts (*p* = 0.008), or have a family history of suicide or suicide attempt (*p* = 0.0004).

Half (50.5%) of patients with documentation confirming a suicide attempt had a service marker for observation versus 10.7% of patients with confirmed self-harm but no documentation of suicide attempt (*p* < 0.001). In contrast, there was no statistically significant difference in observation status between those with (33.0%) and without (23.9%; *p* = 0.15) documented suicidal ideation.

## Discussion

This medical record review determined that the study surveillance definition for ISH injuries had a high PPV (above 80%) for coded cases falling within the proposed surveillance definition in the study population. The study findings also added to evidence regarding a shortcoming in an ISH injury surveillance definition dependent on ICD-10-CM coding: ICD-10-CM codes do not reflect critical clinical information on the intent to die as documented by the providers.(Callahan et al. 2013; Brown et al. 2015; Rockett et al. 2018) According to the ICD-10-CM coding guidelines, the code T14.91 (suicide attempt) is to be assigned only when the mechanism of the intentional injury cannot be determined.(Chan et al. 2016) The code is thus interpreted as “intentional self-harm with unspecified mechanism of injury.” Because of these limitations in ICD-10-CM coding, the study surveillance definition for ISH is unable to distinguish between ISH with and without lethal intent. Our study found that 79 (38.2%) of the ISH injuries in this pediatric sample included documentation by the attending physician indicating the injury was inflicted with an intent to die, a status that cannot be determined using the options available to medical coders. This information is therefore unavailable for epidemiological analysis and policy or program decisions. The analysis also found that use of the suicide attempt code (T14.91) rarely varied from coding guidelines. In only two cases, T14.91 was used in tandem with codes that specified the mechanism of injury.

Past studies examining the quality of ICD-10 coding for ISH and SA in Canadian and Australian ED administrative data have found self-harm events to be largely undercounted (Sveticic et al. 2020; Randall et al., 2017). ISH and SA ICD-10 codes were found to have both low sensitivity and low positive predictive values (PPV) within a sample of both adult and pediatric patients (Sveticic et al. 2020). Our study adds to this literature by assessing these issues using the U.S. version of ICD-10 for morbidity data.

Further investigation is needed to estimate the prevalence of suicide attempts among pediatric patients treated in emergency department settings. A sub-indicator for ISH injuries that accounts for the concurrent presence of a code for suicidal ideation (R45.851) may be informative in epidemiological and policy research, although suicidal ideation alone does not confirm lethal intent.

Examination of ED patients’ discharge status provides insight into the severity of injury and suicidality. Although the majority (73.9%) of patients were discharged home with instructions for outpatient follow-up, over a quarter of cases (26.1%) required admission to a different inpatient facility. Thus, among the patients who were not admitted to inpatient care directly from the ED, approximately one in four cases in the study sample warranted immediate psychiatric or medical management that an outpatient setting could not provide. The decision to admit patients may reflect not only the need for further medical or psychiatric management, but also individual factors such as ability to comply with follow-up instructions, social support, and availability of outpatient resources.

Nuanced challenges may arise from the impulsive nature of self-harm decision-making among adolescent patient populations. The relationship between impulsivity and self-harm behavior is well-documented,(Lockwood et al. 2017) especially among adolescents who report both non-suicidal ISH injury and suicide attempt.(Liang et al. 2014) However, the commonplace nature of impulsive decision-making may complicate the clinician’s assessment. Specifically, adolescent patients’ self-reported history may vary throughout the course of an encounter. Furthermore, adolescents’ feelings of suicidal ideation may fluctuate, exacerbating inconsistent reporting.

### Limitations

This study provides data from a single hospital system where the study cases received care and may not be generalizable to other settings. The percentage of patients admitted to inpatient facilities in this sample is clearly an underestimate because the discharge records for the pediatric patients who were seen in the study hospital ED and admitted to inpatient care in the same hospital are classified as inpatient records in the administrative data system and not included in this study.

The sampling criteria did not capture two pediatric cases in the study ED’s discharge data that included ISH codes. In these cases, an external cause-of-injury code other than ISH was listed in a prioritized field, and an ISH code was listed in a secondary diagnosis field. This finding indicates a slight risk of excluding cases when using standard selection criteria. Our findings are limited to the group of cases with codes that fell within the proposed case definition, not the entire set of adolescent ED cases, and may also have missed cases where no ICD-10-CM code fell within the proposed coding groups. In sum, additional case confirmation studies in different health care settings are needed to evaluate the ability of self-harm codes to identify true cases. Last, given the direct statistical relationship between PPV and population prevalence, the estimated PPVs in this sample may be higher than in hospital systems that care for disproportionately fewer cases of self-harm.(Tenny and Hoffman 2020)

## Conclusion

Our review of pediatric ED visits captured by the study surveillance definition for ISH injuries found a high positive predictive value (88.9%). The medical record review also found that more than one-third of the ISH injuries were suicide attempts. However, the ICD-10-CM coding does not support capture and reporting of ISH injuries with lethal intent, reflecting a longstanding problem with the use of these codes for suicide surveillance. The limitations in the coding system identified by our findings could impede service delivery for this highly vulnerable population. The wording for the code T14.91, suicide attempt, does not correspond to the way the code is used, because its use is restricted to cases where the mechanism of the suicide attempt is not specified in the medical record. Epidemiologists should be made aware of this limitation to avoid interpreting the frequency of T14.91 as a count of records indicating suicide attempt. Further studies are needed to support the development and the validation of surveillance definitions for ISH injuries in ED and inpatient settings among pediatric patients as well as the general population.

## Data Availability

The abstract form used in this study has been included in this submission. Data are not available because of data use agreement restrictions.

## Abbreviations

CDC: Centers for Disease Control and Prevention
ED: Emergency department
HIPAA: Health Insurance Portability and Accountability Act
ICD-10-CM: International Classification of Diseases, 10th Edition, Clinical Modification
ISH: Intentional self-harm
PPV: Positive predictive value
SA: Suicide attempt
